# Efficient modification of λ-DNA substrates for single-molecule studies

**DOI:** 10.1038/s41598-017-01984-x

**Published:** 2017-05-18

**Authors:** Yoori Kim, Armando de la Torre, Andrew A. Leal, Ilya J. Finkelstein

**Affiliations:** 1 Department of Molecular Biosciences and Institute for Cellular and Molecular Biology, The University of Texas at Austin, Austin, Texas 78712 USA; 2 Center for Systems and Synthetic Biology, The University of Texas at Austin, Austin, Texas 78712 USA

## Abstract

Single-molecule studies of protein-nucleic acid interactions frequently require site-specific modification of long DNA substrates. The bacteriophage λ is a convenient source of high quality long (48.5 kb) DNA. However, introducing specific sequences, tertiary structures, and chemical modifications into λ-DNA remains technically challenging. Most current approaches rely on multi-step ligations with low yields and incomplete products. Here, we describe a molecular toolkit for rapid preparation of modified λ-DNA. A set of PCR cassettes facilitates the introduction of recombinant DNA sequences into the λ-phage genome with 90–100% yield. Extrahelical structures and chemical modifications can be inserted at user-defined sites via an improved nicking enzyme-based strategy. As a proof-of-principle, we explore the interactions of *S*. *cerevisiae* Proliferating Cell Nuclear Antigen (yPCNA) with modified DNA sequences and structures incorporated within λ-DNA. Our results demonstrate that *S. cerevisiae* Replication Factor C (yRFC) can load yPCNA onto 5′-ssDNA flaps, (CAG)_13_ triplet repeats, and homoduplex DNA. However, yPCNA remains trapped on the (CAG)_13_ structure, confirming a proposed mechanism for triplet repeat expansion. We anticipate that this molecular toolbox will be broadly useful for other studies that require site-specific modification of long DNA substrates.

## Introduction

Single-molecule imaging and manipulation approaches have greatly expanded our understanding of protein-nucleic acid interactions^1^. More recently, the single-molecule toolkit has grown to include hybrid methods that combine fluorescence imaging (e.g., single-molecule FRET) with force manipulation (e.g., optical or magnetic tweezers) for multi-modal data acquisition^2–4^. Many of these approaches require very long (>10 kb), chemically modified DNA substrates. For example, long DNA substrates are frequently used as flexible, micron-long tethers in optical and magnetic tweezers experiments^4–7^. Moreover, long DNA molecules elevate the biochemical reactions away from glass coverslips and other surfaces, reducing the potential for non-specific interactions^5–7^. DNA derived from bacteriophage λ (λ-DNA) offers several advantages for single-molecule studies. It is a long (48.5 kb) substrate, permitting the observation of protein movement over kilobase-length distances. In addition, synthetic DNA handles can be ligated to the *cosL* and *cosR* ssDNA overhangs. Finally, high-quality recombinant DNA can be purified from lysogenic cells in large quantities, precluding the need to maintain high titer viral stocks.

Site-specific λ-DNA modification strategies generally fall within one of four categories: (i) restriction enzyme cleavage and ligation^4, 8^; (ii) recombinase-mediated modifications^9, 10^; and (iii) insertion of extrahelical structures via oligonucleotide mimics, or (iv) nicking endonuclease (nickase)-based oligo replacement^11–15^. To date, restriction enzyme cleavage and multi-step ligation *in vitro* is one of the most frequently used methods for modifying λ-DNA. However, this approach is technically challenging because multi-step intra-molecular ligation is inefficient. Restriction enzyme-based cloning is also limited due to the few unique restriction sites within the 48.5 kb phage genome. Site-specific recombination *in vitro* is another promising approach for inserting exogenous DNA structures into λ-DNA^9, 10^. However, this requires two unique recombinase sites to be cloned into λ-DNA. The recombinant DNA then needs to be packaged into phage particles, and the virus titer amplified prior to infection of an *E. coli* host. The recombinase enzymes would also need to be produced recombinantly as they are not commercially available. In sum, existing strategies for site-specific λ-DNA modification are either low efficiency or require time-consuming DNA and protein purification.

Introducing site-specific synthetic oligonucleotides and extrahelical structures poses additional challenges in such a long DNA substrate. Such structures are usually inserted via a nicking endonuclease (nickase) based strategy^12, 16–19^. This strategy requires a nicking enzyme to create two or more closely spaced nicks (~10–30 nt apart) on the same strand of the DNA duplex. After nicking, the DNA substrate is annealed with a synthetic oligonucleotide that replaces the short fragments produced by the nickase. However, nickase-based insertion is inefficient for wild type λ-DNA because nickases that produce closely-spaced nicks also cleave hundreds of additional sites on both the top and bottom DNA strands, fragmenting the DNA substrate and reducing the overall yield of full-length DNA molecules. Because this strategy relies on naturally occurring nick sites, it also precludes site-specific incorporation of extrahelical structures at user-specified positions along the DNA substrate.

Here, we develop a method for rapidly modifying and purifying recombinant λ-phage DNA. We use *in vivo* recombineering to target any segment of a lysogenic phage, abrogating the need for restriction sites and ligation. Using this approach, we develop a molecular toolkit for inserting exogenous DNA sequences into the λ-phage genome with >90% efficiency. Site-specific DNA incorporation at three unique sites is confirmed via both ensemble and single-molecule fluorescence assays. We also demonstrate a strategy for inserting non-replicative extrahelical DNA structures at these sites. We explore the utility of these DNA structures by demonstrating site-specific loading of *S. cerevisiae* Proliferating Cell Nuclear Antigen (yPCNA) by the *S. cerevisiae* clamp-loader complex Replication Factor C (yRFC). Our results show that yPCNA can be loaded on a 5′-single stranded DNA (ssDNA) flap, a (CAG)_13_ triplet nucleotide repeat (TNR), and homoduplex DNA. While yPCNA can diffuse freely from the flap and along homoduplex DNA, it remains trapped within the (CAG)_13_ repeat, adding evidence to a model where stationary yPCNA promotes TNR expansion^20–22^. In sum, we anticipate that this molecular toolkit will be broadly useful for both ensemble and single-molecule studies that require site-specific modification of long DNA substrates.

## Results

### A molecular toolkit for modifying λ-phage DNA

**Figure 1:**
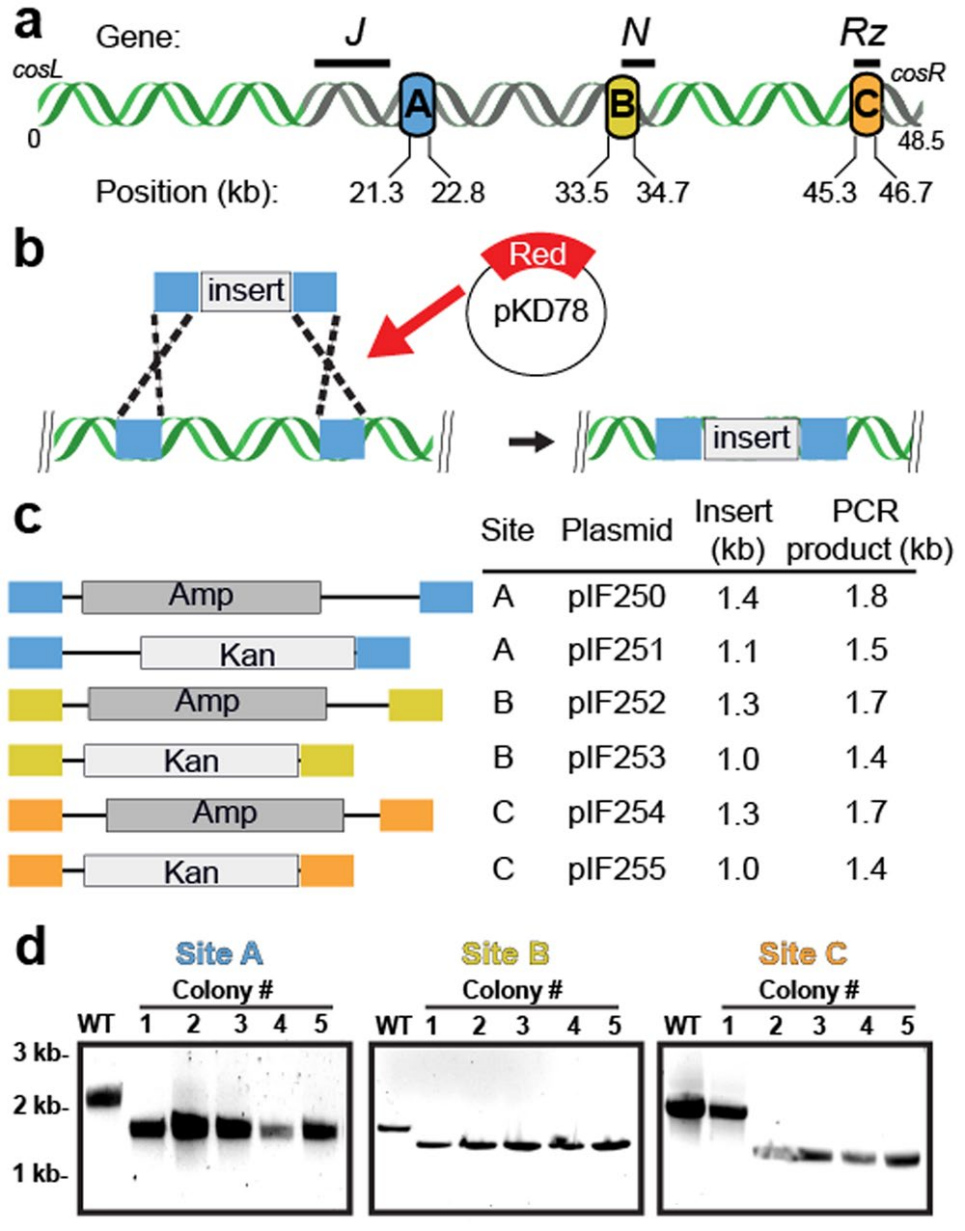
Recombineering at three unique positions within the λ-phage genome. (**a**) Insertion cassettes were designed for three sites targeting dispensable segments of the λ-phage genome (shown in gray). These cassettes are 21.3 kb, 33.5 kb and 45.3 kb away from *cosL* (designated A, B and C, respectively). (**b**) Schematic of the Red-based recombineering. An arabinose-inducible plasmid supplies the three Red genes. (**c**) Cassettes for efficient recombineering into each of the sites shown in (**a**). The cassettes carry an antibiotic resistance gene that is flanked on both sides by ~200 bp of homology to λ-DNA. (**d**) Recombineering efficiency was scored by colony PCR followed by agarose gels analysis. Successful recombineering generates a smaller PCR fragment. We observed 90–100% insertion efficiency at each of the three target sites.

We sought to develop a method that fulfilled three criteria: (i) multiple exogenous DNA sequences can be inserted into λ-phage DNA with nearly 100% efficiency, (ii) the insertion positions are not limited by availability of unique restriction sites, and (iii) chemical modifications and extra-helical structures can be introduced efficiently and with minimal handling. To satisfy the first design criterion, we surveyed the literature to identify two large segments encompassing 37% (17.6  kilobase; kb) of the λ-phage genome that are dispensable for both lytic and lysogenic growth (Fig. 1a). The first dispensable segment is 16 kb long and is between gene products *J* and *N* ^23, 24^. The second 1.6 kb segment, is between gene product *Rz* and the *cosR* end^25^. Next, we chose three sites—designated sites A, B, and C (Fig. 1a)—because they are each separated by ~12 kb (~4 µm) and are thus readily resolved via single-molecule fluorescence microscopy (see below). These three sites provide convenient site-specific modifications within λ-phage DNA.

To satisfy the second design criterion, we developed a series of plasmids for efficient recombineering-based insertion of exogenous DNA into the λ-phage genome. Recombineering is a recombination-based method for genome modification *in vivo* (Fig. 1b)^26, 27^. Recombineering proteins, supplied on an arabinose-inducible helper plasmid, catalyze the insertion of a linear DNA substrate between two homologous sites in the phage genome^28^. The phage is maintained as an inducible lysogen in *E. coli*, precluding the need to amplify and maintain high-titer phage particles^26^. We synthesized a series of plasmids with targeting cassettes for sites A, B, and C (Fig. 1c). The cassettes consist of a user-specified DNA and an antibiotic resistance gene flanked by ~200 bp of homology to the insertion site (see Supplemental Information). Increasing the length of the homologous arms facilitates efficient recombineering^29^. Resistance to an antibiotic marker further selects the desired product. The recombineering reaction efficiency was scored via colony PCR and agarose gel electrophoresis. Each of the three insertion cassettes replaces the native sequence with a ~1–1.5 kb insert (Fig. 1c). As expected, the recombineering efficiency approached nearly 100% at each of the three insertion sites (Fig. 1d, 100%, N = 10/10; 100%, N = 10/10; and 90%, N = 9/10 colonies for sites A, B, and C, respectively). To amplify the recombinant λ-DNA, lytic growth was induced at a non-permissive temperature (>42 °C). Unfolding of the temperature-sensitive repressor (cI857*ind 1*) initiates phage replication and packaging^24, 30^. An additional amber mutation in the S gene (*Sam 7*) delays cell lysis and produces large burst sizes (>200 phage particles per cell), increasing the overall yield of phage DNA^23, 31^. Insertion of the cassette was confirmed via PCR, restriction enzyme digestion, and single-molecule imaging (see below).

**Figure 2:**
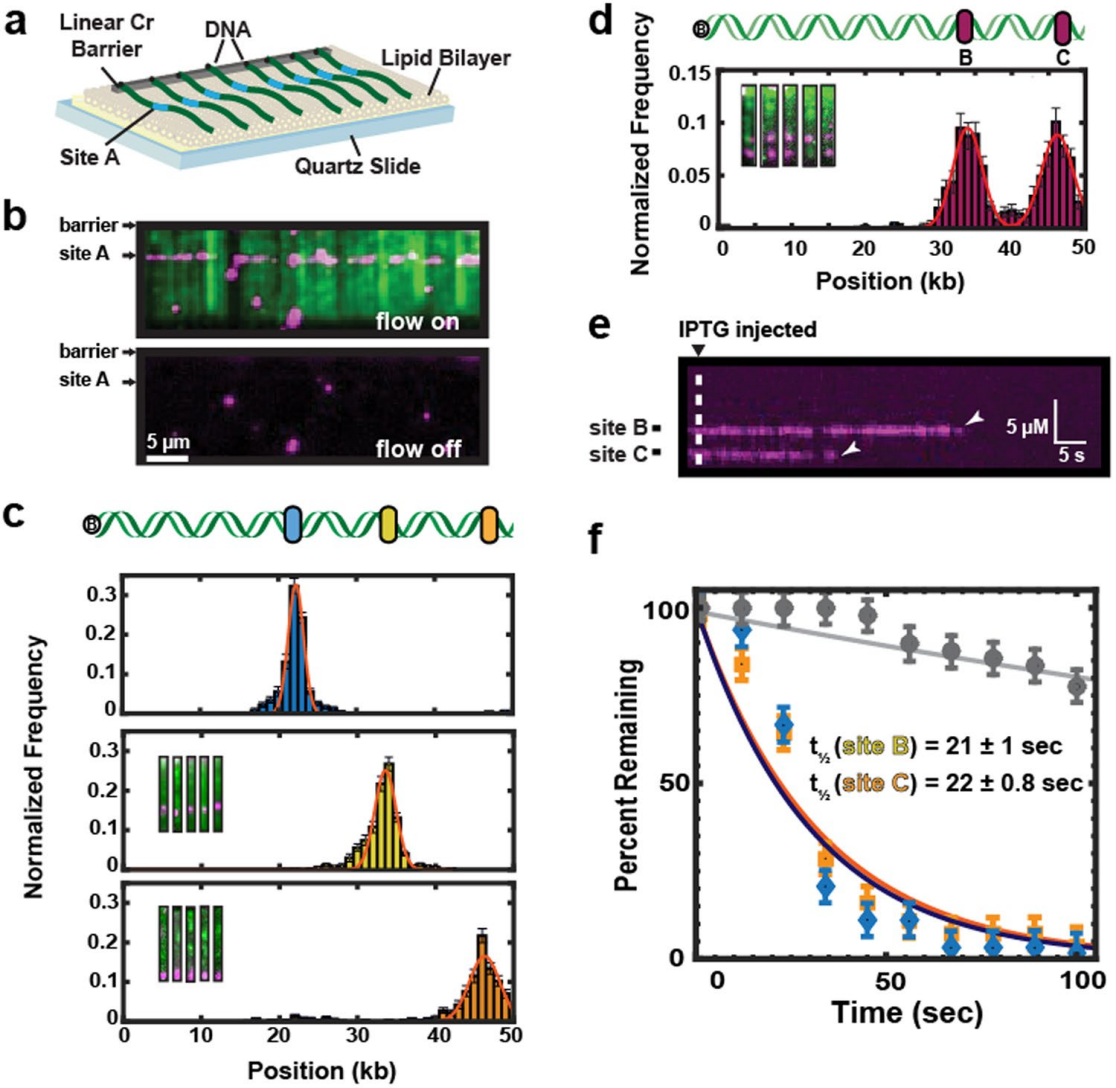
Single-molecule imaging of Lac repressor (LacI) dynamics on recombineered λ- DNA substrates. (**a**) Schematic of a single-tethered DNA curtain assay. DNA molecules (green) are immobilized on a fluid lipid bilayer and arranged at micro-fabricated Chromium (Cr) barriers. One of the insertion sites (site A) is indicated in blue. (**b**) TIRFM image of LacI (magenta) binding to a lac operator inserted at site A on a recombineered λ-DNA substrate (green). (**c**) Binding distributions of LacI to a lac operator sequence inserted at one of the three sites within λ-DNA. Solid lines indicate a Gaussian fit to the data with the indicated mean ± standard deviation. Blue: site A (21.7 ± 1.0 kb, N = 558, blue). Yellow: site B (34.0 ± 1.5 kb, mean N = 538). Orange: site C (46.0 ± 2.3 kb, N = 467). (**d**) Insertion of two lac operator cassettes within a single λ-DNA construct. Inset: single-molecule imaging of the recombinant DNA shows LacI at both positions B & C. Binding histograms show nearly equal occupancy at both lac operator sites. Red line: double Gaussian fit (34.0 ± 2.0 kb and 46.2 ± 2.2 kb, respectively; N = 525). (**e**) As expected, LacI dissociates from both operator sites after injection of 0.4 mM Isopropyl β-D-1-thiogalactopyranoside (IPTG). (**f**) Quantification of the LacI lifetimes upon IPTG injection. The solid lines are single-exponential fits to the data (blue diamonds, t_½_ = 21 ± 1.0 sec, N = 63, and orange squares, t_½_ = 22 ± 0.8 sec, N = 56 at positions B & C, respectfully). The solid gray line is the single-exponential fit of the LacI lifetime data, in the absence of IPTG (grey circles, t_½_ = 327 ± 10 s, N = 49). The error bars indicate the standard deviation obtained via bootstrap analysis.

As a first proof-of-principle, high-throughput DNA curtains were used to measure the binding positions of Lac repressor (LacI) proteins on recombinant λ-DNA harboring a high-affinity Lac operator sequence. In this assay, a fluid lipid bilayer is deposited on the surface of a micro-patterned flowcell^32–34^. Biotinylated DNA is immobilized on the lipid bilayer via a biotin-streptavidin linkage and buffer flow is used to organize hundreds of DNA molecules at chromium (Cr) diffusion barriers (Fig. 2a)^32, 34^. LacI was incubated with the DNA and fluorescently labeled with anti-FLAG antibody-conjugated quantum dots (QDs). The DNA was stained with a fluorescent intercalating dye (YOYO-1) and 89% (N = 89/100) of the DNA molecules were full-length (Fig. 2b). This measurement likely underestimates the percentage of full-length DNA because intercalating dyes induce photodamage and accelerate the accumulation of broken molecules. As expected, LacI bound specifically to the recombinant operator site (Fig. 2c) at each of our three target sites^35^. These results demonstrate that high quality recombinant λ-DNA provides an ideal substrate for single-molecule imaging.

Next, we developed a strategy for inserting multiple recombinant DNA sequences—each flanked by a different antibiotic cassette—into the same λ-DNA substrate. All colonies (N = 10/10) harbored both insertion cassettes, as scored by colony PCR. To confirm the quality of these DNA substrates for single-molecule experiments, we imaged LacI binding to DNA curtains harboring a Lac operator in both sites B & C. As expected, 92% (N = 483/525) of all LacI molecules bound to either one of the two insertion sites (Fig. 2d). The broad LacI binding distributions largely stem from experimental uncertainty in the precise surface tethering of DNA molecules (Fig. 2c and d). To construct binding histograms, we assume that all DNA molecules are tethered at the center of the diffusion barriers, which are ~1–1.5 µm wide^32^. When compiling binding distributions from hundreds of such DNA molecules (spanning several flowcells), this approximation results in an apparent broadening of the resulting histogram. This broadening has also been observed for both site- and sequence-specific DNA-binding proteins in similar flow-stretched assays^36–40^. We further confirmed that LacI is bound to the operator site by injecting 1 mM IPTG into the flowcell. IPTG caused rapid dissociation of all LacI molecules with a half-life of 21 ± 1.0 seconds (N = 63; error indicates 95% C.I.) for site B and 22 ± 0.8 seconds (N = 56) for site C (Fig. 2e and f). In contrast, the half-life of LacI in the absence of IPTG was 327 ± 10 seconds (N = 49). We conclude that recombinant λ-DNA can be rapidly modified at multiple sites for single-molecule studies.

### Insertion of modified oligonucleotides into λ-DNA

**Figure 3:**
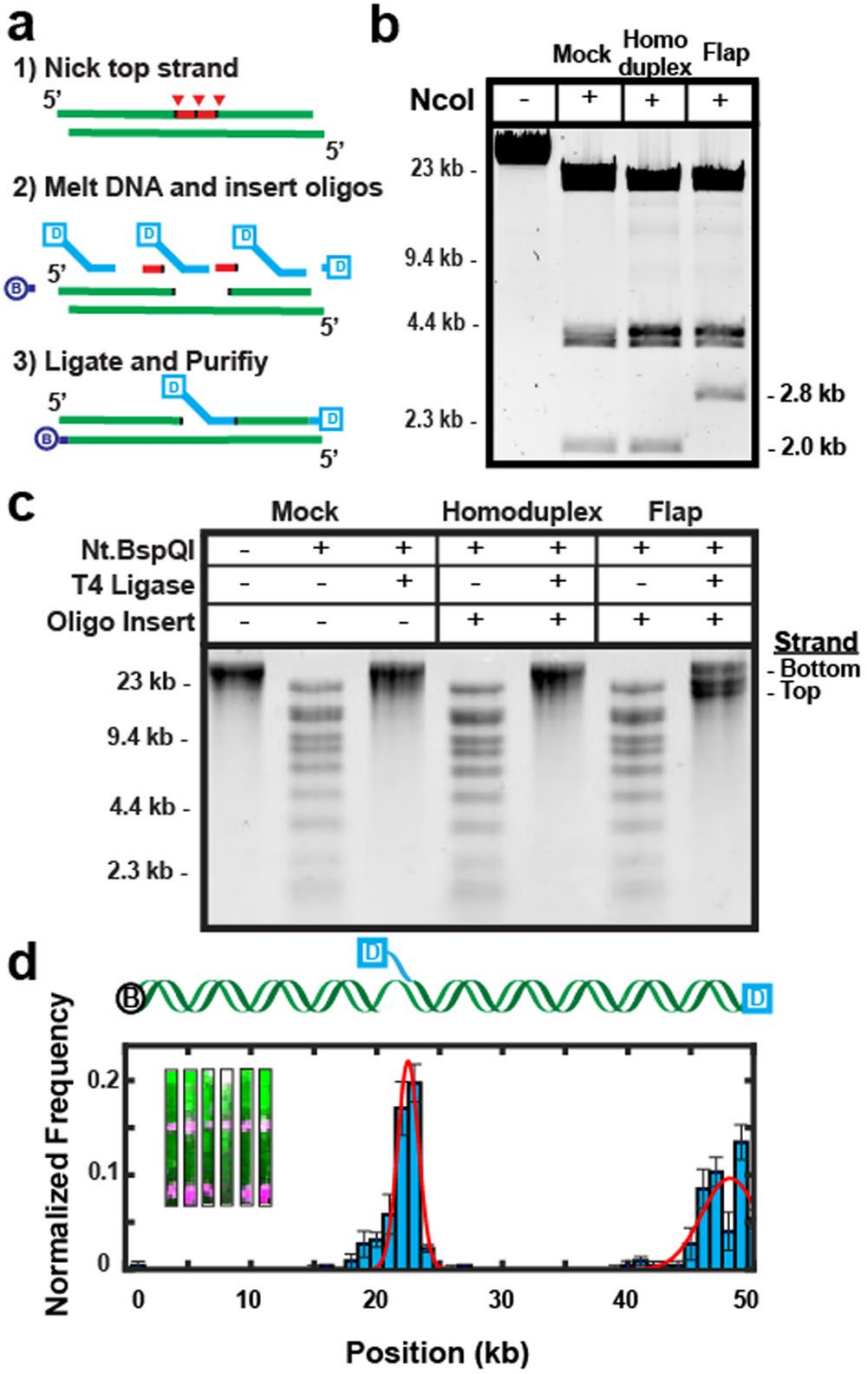
Construction of DNA substrates containing extrahelical structures. (**a**) Schematic of nickase-mediated insertion of a synthetic oligonucleotide (Digoxigenin (dig)-labeled 5′-flap). (**b**) Diagnostic digest of a recombineered λ, demonstrated here by the 2.8 kb band in lane 4 which is digested into two smaller bands in lanes 2 and 3 (bottom band not shown). (**c**) Denaturing alkaline agarose gel of three separate reactions (a “mock-treated” DNA where the nicked DNA is replaced with a complementary oligo, a homoduplex DNA, and a 5′-ssDNA flap) to determine ligation efficiency. The last lane shows a larger band (bottom strand) and two smaller bands (top strand) that are created upon insertion of the flap. (**d**) Top: schematic of a DNA substrate containing a dig-labeled 5′-ssDNA flap and a dig-terminated free DNA end. Bottom: binding distribution of anti-dig quantum-dots to this DNA substrate. Solid lines indicate a double Gaussian fit to the data with the indicated mean ± standard deviation (5′-ssDNA flap: 22 ± 0.8 kb; 3′-DNA end: 47.5 ± 2 kb; N = 225). Insets: representative DNA molecules (green) labeled with quantum dots (magenta) at position A and at the free DNA ends.

Single-molecule studies frequently require the insertion of modified bases or fluorophores at precise positions along the DNA substrates. We developed an improved nicking-enzyme based strategy for inserting synthetic oligonucleotides at precise positions along λ-DNA (Fig. 3a). We designed a nicking cassette that includes three consecutive BspQI recognition sequences separated by 12–13 bp spacers (see Supplemental Information). The DNA is nicked with Nt.BspQI (nickase) enzyme that creates closely spaced discontinuities along one of the two DNA strands. λ-DNA harbors ten additional widely spaced BspQI recognition sequences, thereby limiting nicks and double-strand breaks along the rest of the DNA substrate. The nicked DNA is heated to liberate short single-stranded DNA fragments. A synthetic oligonucleotide is inserted into the backbone by annealing and ligation^11^. After nicking with Nt.BspQI and heating, the gapped DNA is slowly cooled with a 100-fold excess of the desired insertion oligo. The annealed and ligated λ-DNA is further purified by gel filtration to remove enzymes and excess oligonucleotides. Conversion of restriction sites (NcoI, NotI) to single-stranded DNA within the nicking cassette fascilitates rapid quantification of the insertion efficiency via restriction digest mapping.

We validated this approach by inserting extrahelical structures into a nicking cassette that was cloned into λ-DNA using the recombineering strategy described above. We first inserted a 5′-ssDNA flap oligonucleotide that annealed to 26 nt of the gapped ssDNA. Insertion of the flap oligonucleotide abolishes a single NcoI site within the cassette, converting two small NcoI DNA fragments (2.0 kb + 0.8 kb) to a larger 2.8 kb band on a native agarose gel (Fig. 3b). We saw 100% cleavage of the flap-proximal NcoI site with both untreated homoduplex DNA and with a complementary phosphorylated oligonucleotide that perfectly replaced the excised cassette (i.e., mock-treated substrate). However, only ~1% of the DNA was NcoI-sensitive after 5′-ssDNA flap incorporation. Next, alkaline agarose gel electrophoresis was used to assay the quality of the ligated DNA substrate (Fig. 3c). The 48.5-kb DNA runs as a single large band on a denaturing gel. Nicking with Nt.BspQI produces a distinct ladder of bands, which are removed after ligation. As expected, ligating the nicked DNA substrate with a complementary phosphorylated oligonucleotide (mock-treated DNA) produces a substrate that is indistinguishable from homoduplex λ-DNA. Importantly, adding the 5′-flap oligonucleotide produces a DNA substrate with three distinct bands on the alkaline agarose gel; the full-length bottom strand and the 21.3 kb and 26.7 kb discontinuous top strands (Fig. 3c). The quality of the DNA substrate and site-specific flap incorporation was also evaluated via single-molecule fluorescence imaging (Fig. 3d). Single-tethered DNA curtains were assembled with λ-DNA that has a biotin on the *cosL* end and a digoxigenin on both the inserted flap and the *cosR* end. Digoxigenin was fluorescently labeled via anti-digoxigenin-functionalized QDs. The fluorescent signal was localized to the flap and DNA ends (Fig. 3d, inset). A histogram of the binding positions showed two peaks, one at the distal DNA end and a second at site A, where the ssDNA flap was incorporated (Fig. 3d). Importantly, 82% (N = 82/100) of the fluorescently stained DNA molecules were full-length, confirming that the DNA was not fragmented after the oligo insertion reaction. In sum, our ensemble biochemical and single-molecule results confirm efficient and site-specific incorporation of synthetic oligonucleotides into full-length λ-DNA.

### Observing *S. cerevisiae* PCNA (yPCNA) dynamics on modified DNA substrates

To further demonstrate the utility of modified λ-DNA substrates for single-molecule studies, we determined how yRFC loads yPCNA on various DNA structures. RFC loads PCNA by using the energy of ATP hydrolysis to open and close the clamp ring^41–43^. Although RFC preferentially loads PCNA on primer-template junctions, it can also catalyze loading on nonspecific homoduplex dsDNA. RFC has also been proposed to load PCNA on extrahelical triplet nucleotides repeats (TNRs), ultimately driving TNR expansion^20–22^. However, nearly all prior biochemical studies have used a truncated yRFCΔN, where the DNA-binding patch of Rfc1 has been deleted^44^. This is because a dsDNA-binding motif within the large RFC subunit (Rfc1) binds dsDNA non-specifically, complicating ensemble biochemical experiments^45^. Therefore, we first assayed yPCNA loading by the more physiologically relevant full length wild type (wt) yRFC^46^.

**Figure 4:**
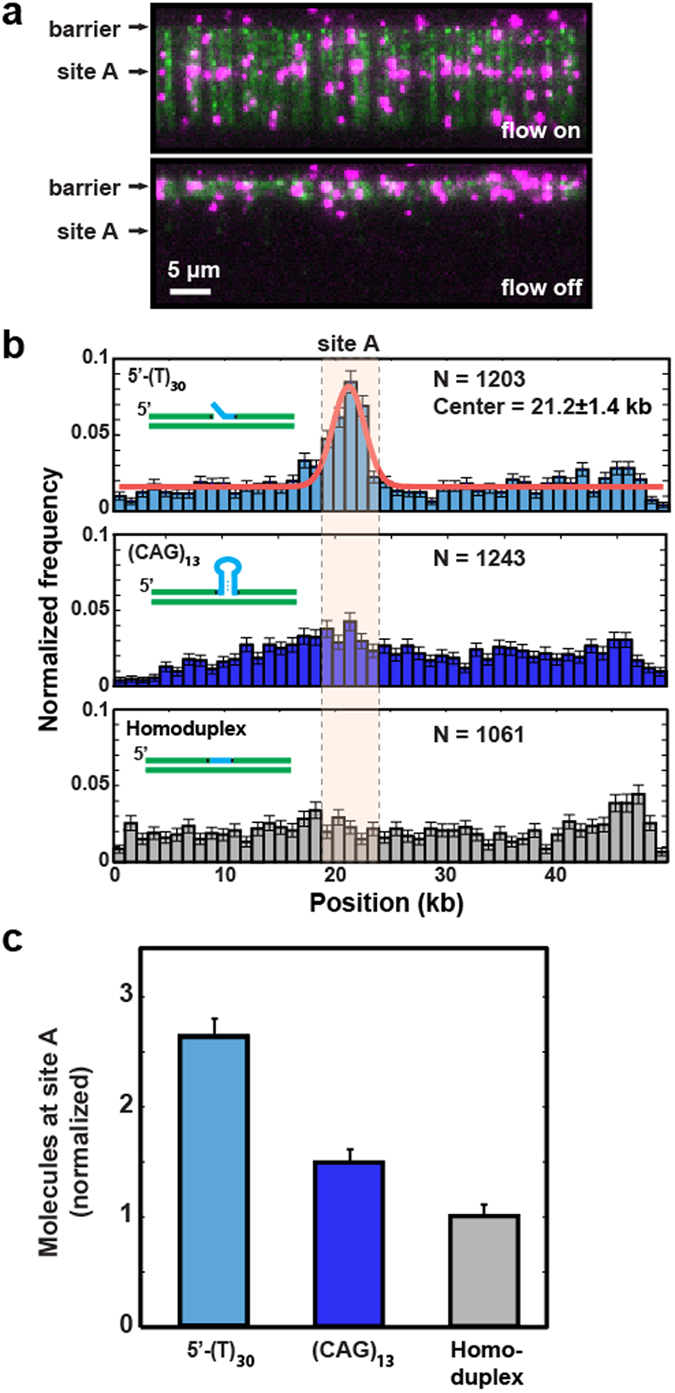
yRFC loads yPCNA on various extrahelical DNA structures. (**a**) Fluorescent images of yPCNA on DNA substrates with a 5′-ssDNA flap inserted at site A. Top: with buffer flow, bottom: without flow. DNA is stained with YOYO-1 (green) and yPCNA is labeled with anti-FLAG antibody conjugated QDs (magenta). Stopping buffer flow retracts both yPCNA and DNA to the Cr barrier, confirming that yPCNA is bound to the DNA and is not on the surface (bottom panel). (**b**) Binding distribution histogram of yPCNA/yRFC on λ-DNA molecules containing a 5′-ssDNA flap (top), (CAG)_13_ repeat (middle), and homoduplex DNA (bottom). Red line: fit to a Gaussian distribution (center = 21.2 kb ± 1.4 kb; st. dev.). Pink box: a 5 kb window that captures 99% of all yPCNAs at site A (see text for details). (**c**) The mean number of yPCNA molecules loaded at site A (within the pink box), as determined by bootstrap analysis of the histograms in (**b**). Error bars represent the st. dev. of the mean from the bootstrap analysis.

We first explored the relative specificity of yPCNA loading onto DNA substrates harboring a 30-nt 5′-ssDNA flap, a (CAG)_13_ repeat, or homoduplex dsDNA (Fig. 4). For fluorescent labeling, yPCNA was over-expressed with a triple FLAG epitope tag on the N-terminus, which is far away from the DNA binding site^42, 47^. The slowly hydrolyzable ATPγS was used in the pre-incubation and loading reactions to capture the yRFC-yPCNA complex at the loading site. ATPγS prevents yPCNA ring closing and release from yRFC^42, 44, 48^. yPCNA was fluorescently labeled *in situ* with anti-FLAG functionalized quantum dots (QDs), as described previously for human RFC-PCNA (hRFC-hPCNA)^49^. We confirmed that yPCNA loading was yRFC-dependent (not shown). The yRFC(ATPγS)-yPCNA complex concentration was also titrated down to yield one or fewer fluorescent yPCNAs per DNA molecule.

As expected, yRFC preferentially loads yPCNA at the 5′-ssDNA flap relative to homoduplex DNA, which is consistent with prior studies (Fig. 4a and b)^50^. Turning off buffer flow retracted both the DNA and yPCNA to the Cr barrier, confirming that yPCNA was loaded on the DNA (Fig. 4a, bottom). We also observed mild enrichment of yPCNA at a (CAG)_13_ repeat (Fig. 4b, middle) relative to homoduplex DNA (Fig. 4b, bottom). To better characterize the loading efficiency on these structures, we calculated the relative enrichment of yPCNA in a 5-kb window spanning the nicking cassette on the three recombinant DNA molecules (Fig. 4c). This window was selected because it captures ~99% (~3 standard deviations of the Gaussian fit in Fig. 4b, top) of all site-specifically bound yPCNA molecules. Loading at the 5′-ssDNA flap showed 2.7-fold enrichment over homoduplex DNA, whereas loading at the (CAG)_13_ was 1.5-fold higher than homoduplex DNA. Because the enrichment at (CAG)_13_ was very modest, we also tested the yPCNA binding to (CAG)_13_ inserted at site B and at site A with a flipped tethering geometry (Fig. S1). The same mild yPCNA enrichment was observed at the expected (CAG)_13_ position in all three DNA substrates (Fig. S1). Moreover, this enrichment was statistically significant (p-value: 1 × 10^−4^) when compared against all possible 5 kb windows across the entire 48.5 kb DNA substrate (see Statistical Methods and Fig. S1c). We conclude that wt yRFC has a mild preference for loading yPCNA onto (CAG)_13_ repeats relative to homoduplex DNA.

**Figure 5:**
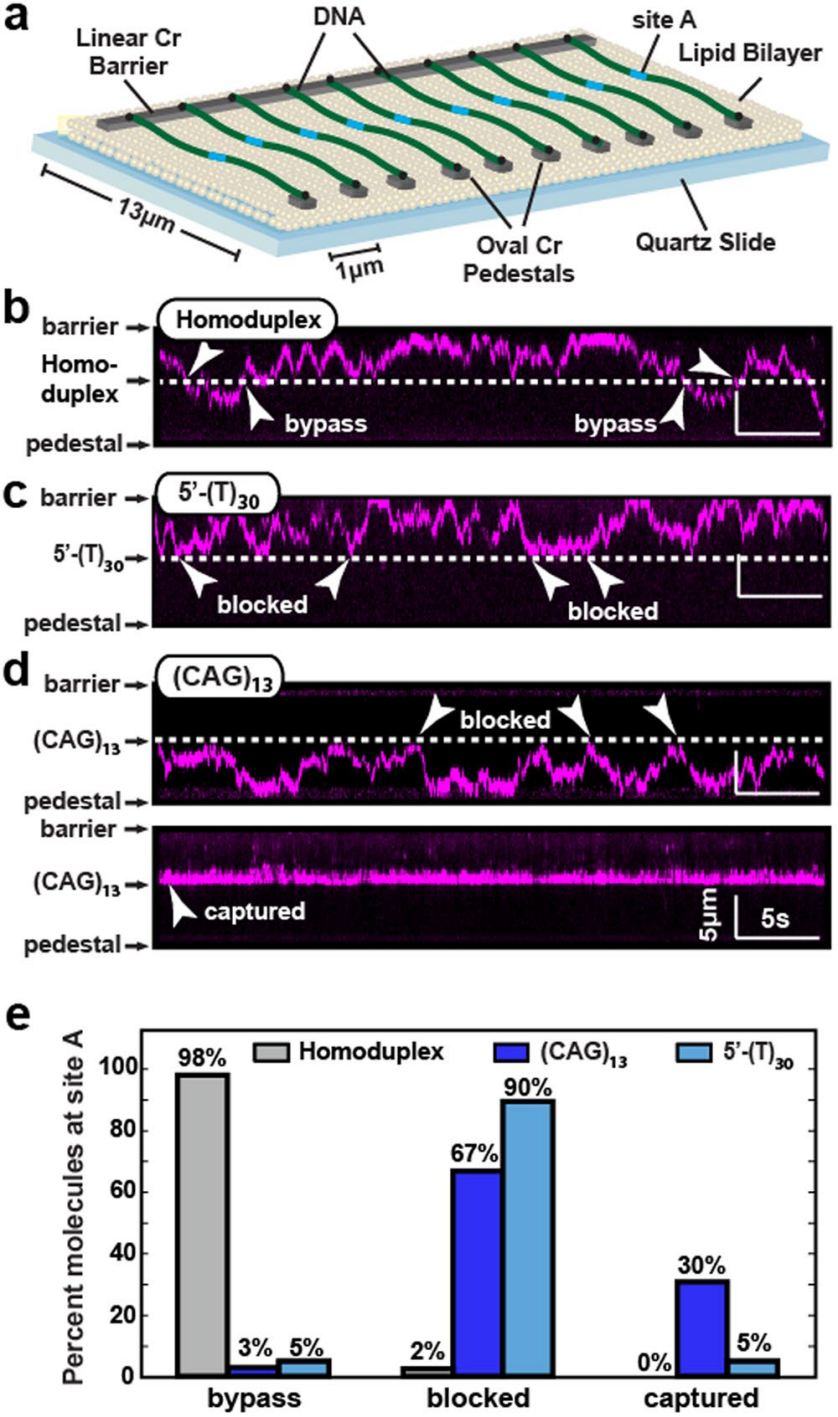
yPCNA diffusion on λ-DNA containing various extrahelical structures. (**a**) An illustration of the double-tethered DNA curtains. Chromium (Cr) pedestals are coated with anti-digoxigenin antibodies, and buffer flow is used to immobilize the dig-labeled λ-DNA between the linear barriers and Cr pedestals. Kymograph of diffusing yPCNA molecule on DNA substrates with (**b**) homoduplex DNA (**c**) a 5′-ssDNA flap or a (CAG)_13_ repeat (**d**). The characteristic changes in diffusion behavior of yPCNA are indicated with arrows, and the dashed lines indicate site A. (**e**) Percentage of molecules showing either bypass, blocked, or captured behavior at site A. At least 35 DNA molecules were analyzed and classified into each of three categories (N = 40, 36, and 40 for the flap, (CAG)_13_, and homoduplex DNA substrates).

PCNA has a 3.4 nm-diameter inner opening that is wider than the diameter of B-form DNA^42, 45^. This inner opening facilitates sliding past small mismatches and bulges, but may be blocked by larger extrahelical structures^50^. To directly test this hypothesis, we used double-tethered DNA curtains to observe yPCNA diffusion on various DNA substrates (Fig. 5a). In this assay, one end of the DNA was labeled with a biotin and the second end was labeled with a digoxigenin (dig). Pedestals that were located 13 µm away from the diffusion barriers were decorated with anti-dig antibodies. The antibody-coated pedestals provide a second attachment point for dig-labeled DNA molecules^32, 34^. The DNA molecules that are tethered between the barriers and pedestal remain fully extended without the need for additional buffer flow (Fig. 5a). As buffer flow can bias protein diffusion on DNA, double-tethered curtains provide an ideal platform for monitoring yPCNA dynamics. We first observed yPCNA diffusion on homoduplex DNA. For these experiments, ATP was added to the yRFC-yPCNA pre-incubation reaction. ATP hydrolysis catalyzes closing of the yPCNA ring and release of yRFC from DNA. To ensure complete yRFC removal from DNA, the flowcells were further washed with BSA buffer containing 300 mM NaCl. Following yRFC removal, the yPCNA molecules diffused freely over the entire length of the double-tethered DNA curtains (Fig. 5b).

Ring-like proteins can diffuse on DNA via a combination of two different modes: 1D sliding and/or hopping. During 1D sliding, the protein tracks the helical backbone of the dsDNA. In contrast, hopping is characterized by a series of correlated microscopic detachment and reattachment events. To differentiate between sliding and hopping, we measured yPCNA diffusion coefficients at increasing ionic strengths (Fig. S2). A higher ionic strength increases electrostatic screening between a protein and DNA, thereby reducing the fraction of time that a protein is in contact with the DNA. This results in increased diffusion coefficients at higher ionic strengths. yPCNA diffusion was weakly dependent on the ionic strength, in agreement with a previous study with hPCNA (Fig. S2)^49^. The ionic strength-dependent change in diffusion coefficients was 0.33 ± 0.04 µm^2^ sec^−1^ mM^−1^ and 0.25 ± 0.06 µm^2^ sec^−1^ mM^−1^ for hPCNA and yPCNA, respectively. This result indicates that like hPCNA, yPCNA also moves predominantly by 1D sliding along the helical pitch of the DNA substrate^49^.

We next monitored yPCNA diffusion on DNA harboring a 5′-ssDNA flap or a (CAG)_13_ repeat (Fig. 5c,d). yPCNA can approach the flap from one of two orientations: (i) in the *cosL* → *cosR* direction yPCNA encounters a 4-nt ssDNA gap prior to the flap and (ii) in the *cosR* → *cosL* direction yPCNA encounters the 5′-T_30_ tail. Out of 40 yPCNA molecules 90% (N = 36/40) were blocked by the flap in either orientation (N = 18 for *cosL* → *cosR* and N = 18 for *cosR* → *cosL*). The remaining molecules were either captured at the nickase cassette (5%, N = 2/40) or directly bypassed (5%, N = 2/40) the flap structure. This observation may stem from incomplete re-ligation of the flap oligo at this site. These data suggest that the flap is too large to be accommodated within the yPCNA pore. The (CAG)_13_ repeat also blocked yPCNA that was loaded on homoduplex DNA upstream or downstream of site A (Fig. 5d,e). Remarkably, we also observed that 30% (N = 11/36) of the yPCNA molecules loaded directly at the (CAG)_13_ were completely stationary (Fig. 5e). Moving the (CAG)_13_ repeat to a different site in the DNA substrate also moved the location of the stationary yPCNA molecules (Fig. S3a,b). The distribution of freely diffusing, stationary, and (CAG)_13_-blocked yPCNAs was statistically indistinguishable across all three (CAG)_13_-containing DNA substrates (Fig. S3c). We conclude that yPCNA diffusion is blocked by large extrahelical structures and that yRFC loads yPCNA within the (CAG)_13_ repeat. This result is consistent with a mechanism where TNR-bound PCNA interacts with DNA mismatch repair factors to promote TNR expansion^20^.

## Discussion

Here we developed a recombineering-based molecular toolbox for rapid and site-specific incorporation of exogenous DNA sequences and structures in multiple positions in λ-DNA. In this work, 1–1.5 kb cassettes were inserted at three positions along the λ-DNA. Both longer and shorter modifications are also possible, provided that dispensable segments of the phage genome are targeted for modification (Fig. 1). For such applications, we developed a series of ampicillin- and kanamycin-resistant cassettes that can accommodate large insertion fragments that are flanked by 200 bp of homology to three distinct sites within the phage DNA. These markers facilitate positive selection, ultimately yielding nearly 100% recombination efficiency. If required, the drug markers can be removed by flanking the cassettes with FRT sites and inducing Flp recombinase *in vivo* ^26, 28^. Moreover, the DNA substrate may be shortened or expanded, provided that the λ-DNA remains within 78–105% (38 to 53 kb) the length of the wild-type phage genome. This requirement is essential for efficient packaging of the DNA into phage capsids^24^. This flexibility guarantees that both large and small insertions can be made within the phage genome.

We regularly achieved 90–100% recombineering efficiency using PCR constructs directed towards one of three unique sites within the λ-phage genome. However, we also noticed that deviating from a few best practices could produce low yields or off-target insertion products. Cryopreserving lysogens with the recombineering helper plasmid resulted in high off-target recombination rates. Substantially higher yields were achieved when the lysogens were freshly transformed with the helper plasmid and used immediately for Red system induction in mid-log cells (at an OD_600_ ~ 0.5 or below). Additionally, recombineering efficiency was higher when the PCR products were both DpnI-treated to remove residual template DNA and gel purified prior to electroporation. After electroporation, a minimum 4-hour outgrowth should be allowed before plating the cells on antibiotic selective plates. Finally, recombineering at site A yielded slow-growing cells that required an overnight outgrowth in LB and up to two days for colonies to appear on antibiotic selective plates. Following these simple guidelines routinely resulted in highly specific modification at the desired locus.

We also demonstrated an improved nickase-based strategy for incorporating extrahelical structures at defined positions along the DNA duplex. Because this approach requires two or more closely spaced nicks, it is most commonly used with plasmid-based modifications^11, 16, 17^. This approach is less suitable for wild type λ-DNA because it requires nicking enzymes that cleaves the substrate at hundreds of sites on both strands, leading to double-stranded breaks and low yields of full-length DNA^16, 17^. Our approach refines the previously published strategies in four key ways: (i) a nicking cassette can be inserted into any dispensable segment of the genome, (ii) nicking at three adjacent sites exposes 39 nt of single stranded DNA, providing sufficient space to insert large oligonucleotides (e.g., long ssDNA gaps, RNA-DNA structures, and other chemically modified oligonucleotides), (iii) using a nickase with few recognition sites minimizes damage to the DNA substrate, and (iv) incorporation efficiency can be rapidly scored via digestion with one of several restriction enzymes.

Using this approach, we demonstrated that yRFC preferentially loads yPCNA on a 5′-ssDNA flap and a (CAG)_13_ repeat relative to homoduplex DNA. yPCNA can diffuse on homoduplex DNA but is blocked by both DNA structures. These observations suggest that yPCNA cannot simultaneously accommodate both the homoduplex DNA track (2 nm diameter for B-form DNA) and the additional DNA structures within its inner ring. Molecular dynamics simulations and ensemble biochemical studies suggest that homopolymeric poly-T oligonucleotides occupy an ensemble of extended structures (e.g., 2.7 nm end-to-end distance for a (T)_12_ oligonucleotide; we used a (T)_30_)^51^. Similarly, structural and biochemical studies have established that relatively short (CNG)_n=3–10_ repeats are in a hairpin-stem structure with mismatched A**·**A base pairs that are flanked by two Watson-Crick G**·**C base pairs^52–55^. However, above a critical threshold of trinucleotide repeats, (CNG)_n>11_, the oligonucleotide transitions from a simple hairpin–stem model to bis- (or multi-branched) hairpin-like structures and bulged out ssDNA loops^53, 55^. This dynamic, multi-branched structure likely explains why a (CAG)_13_ repeat acts as both a loading site and a barrier to yPCNA diffusion. Finally, yPCNA that is directly loaded onto the (CAG)_13_ is trapped at the lesion. These results shed light on PCNA diffusion dynamics and further highlight the utility of this molecular toolkit for both single-molecule and ensemble biochemical studies.

## Materials and Methods

### Proteins and DNA

FLAG-epitope labeled LacI was purified from BL21(DE3) as described previously^56^. Biotinylated anti-FLAG M2 antibodies were purchased from Sigma and streptavidin-conjugated quantum dots (QDs) were purchased from Life Technologies. All oligonucleotides were ordered from IDT and are summarized in Table S1. Plasmids containing an antibiotic cassette flanked by two 200-basepair segments that are homologous to the λ-phage genome were custom synthesized by BioMatik. Plasmid maps and sequences are available via Benchling (https://benchling.com/ifinkelstein). All plasmid modifications were carried out using the Q5 inverse PCR mutagenesis kit (NEB).

### *S. cerevisiae* Replication Factor C (yRFC)

The plasmids pLant2b-RFC-AE and pET11-RFC-BCD were kindly provided by Manju Hingorani^46^. The two plasmids were co-transformed into BL21(DE3) ArcticExpress cells (Agilent). A single colony was inoculated into 680 μL of LB and grown overnight at 37 °C. 200 μL of these cells were then seeded into 2 L LB with 50 μg mL^−1^ kanamycin and 50 μg mL^−1^ carbenicillin. The culture was grown at 30 °C to an OD_600_ ~ 0.6, cooled on ice with swirling to 16 °C and induced with 0.5 mM IPTG. Induction continued for 16 hours at 12 °C. Cells were harvested by centrifuging for 15 minutes (3,300 RCF at 4 °C) and resuspended in 20 mL of lysis buffer (30 mM HEPES [pH 7.5], 0.25 mM EDTA, 5% (v/v) glycerol). The resuspended cell paste were either frozen in liquid nitrogen and stored at −80 °C, or prepared for lysis by adding 250 mM NaCl, 1 mM phenylmethanesulfonyl fluoride (PMSF, Sigma-Aldrich), and 1x HALT protease inhibitor (Thermo-Fisher). Cells were lysed in a homogenizer (Avestin), and centrifuged (140,000 RCF, at 4 °C) for 35 minutes. A home-made 7 mL SP FF column (resin from GE Healthcare) was equilibrated with 35 mL of buffer A (30 mM HEPES [pH 7.5], 0.25 mM EDTA, 5% (v/v) glycerol) + 200 mM NaCl and the clarified lysate was loaded onto the equilibrated column at 0.5 mL min^−1^. The column was then washed with 35 mL of buffer A + 250 mM NaCl and the protein was eluted with a 100 mL gradient to buffer B (30 mM HEPES [pH 7.5], 0.25 mM EDTA, 5% (v/v) glycerol, 1 M NaCl) at 1 mL min^−1^. yRFC-containing fractions were pooled and developed through a 1 mL Q HP column (GE Healthcare) pre-equilibrated with buffer A + 100 mM NaCl. The combined sample from the SP column was loaded onto the Q column at a rate of 0.7 mL min^−1^. The column was washed with 10 mL of buffer A + 110 mM NaCl at 0.8 mL min^−1^, and then eluted with an 18 mL gradient into buffer B at 0.8 mL min^−1^. The eluted Q column fractions were analyzed on a 10% SDS-PAGE gel, and the fractions containing yRFC were combined, frozen in liquid nitrogen, and stored at −80 °C. The yRFC concentration was determined by comparison to a BSA titration curve using SDS-PAGE.

### Proliferating Cell Nuclear Antigen (PCNA)

A plasmid expressing *Saccharomyces cerevisiae* His_6_-PCNA was kindly provided by Francisco Blanco^57^. A triple FLAG epitope was introduced at the N-terminus via inverse PCR mutagenesis (NEB) using primers YK_PCNA01 and YK_PCNA02 (see Table S1) to generate pIF105. pIF105 was transformed into BL21(DE3) codon plus RIL cells. A colony was inoculated into 30 mL LB with 50 μg mL^−1^ kanamycin and 34 μg mL^−1^ chloramphenicol, and grown overnight at 37 °C. Ten mL of the overnight culture were seeded into 1 L LB and grown in the presence of both antibiotics. When the culture reached an OD_600_ ~ 0.5, 0.8 mM IPTG was added and induction continued at 37 °C for 4 hours. Cells were harvested by centrifugation at 3,000 RCF for 10 minutes, and resuspended in 50 mL lysis buffer (50 mM Tris-HCl pH 7.6, 150 mM NaCl, 0.5 mM TECP, 10% (v/v) glycerol) with 1x HALT protease inhibitor. Cells were lysed by sonication on ice and centrifuged at 95,000 RCF for 30 minutes. Imidazole was added to the supernatant to a final concentration of 30 mM. A 5 mL HisTrap HP column (GE Healthcare) was pre-equilibrated with 50 mL lysis buffer and the lysate was loaded onto the column and washed with 50 mL of Ni-buffer (50 mM Tris-HCl [pH 7.6], 150 mM NaCl, 10% glycerol (v/v), 30 mM imidazole). PCNA was eluted with a ~110 mL gradient to Ni-buffer +500 mM imidazole over 120 minutes. PCNA-containing fractions were identified via 12% SDS-PAGE, dialyzed into storage buffer (20 mM Tris-HCl [pH 7.6], 100 mM NaCl, 10% (v/v) glycerol, 1 mM DTT) and concentrated using a centrifugal filter (10 kDa Amicon, Millipore). Small aliquots were frozen in liquid nitrogen and stored at −80 °C.

### Recombineering λ-phage lysogens

Red-based *in vivo* recombination was used to construct recombinant λ-phage DNA^26, 58^. First, a lysogen was created by packaging λ-phage DNA (cI857*ind* 1 *Sam* 7; NEB) into empty phage particles (MaxPlax Lambda Packaging Kit, EpiCentre). The packaged phage was used to infect *E. coli* strain 7723 and lysogens were identified via colony PCR and sensitivity to growth at a restrictive temperature (42–45 °C). The resulting cells (IF189) were transformed with pKD78, which harbors the Red recombineering system under the control of an arabinose-inducible promoter^28^. The insertion cassettes were PCR amplified from helper plasmids (Fig. 1) with custom primers (AD027 and AD028 for site A, AD012 and AD013 for site B, or AD016 and AD017 for site C; see Table S1) using *Taq* DNA polymerase (NEB# M0320S). PCR reactions were treated with 1 U DpnI (NEB #R0176s) at 37 °C for 1 hour and purified by gel extraction (Qiagen Kit #28704) to remove residual plasmid template, which can create false positives during recombineering^26^. The gel-extracted DNA was re-suspended in Milli-Q water to a final concentration of 100–150 μg μL^−1^ and used as the targeting DNA for the recombineering reaction.

Fresh electrocompetent cells were prepared for every recombineering reaction. A 5 mL LB culture of strain IF189 (λ-lysogen) transformed with pKD78 was grown overnight at 30 °C in the presence of 10 μg μL^−1^ chloramphenicol. The following day, 350 µL of cells were used to inoculate a fresh 35 mL culture of LB containing the same concentration of antibiotic. When the cells reached an O.D._600_ ~ 0.5, the Red recombinase system was induced by adding 2% L-arabinose (GoldBio) and incubated for an additional 1 hour at 30 °C. Cells were harvested at 4,500 RCF for 7 min, washed three times in ice-cold Milli-Q H_2_O, and finally resuspended in 200 µL of H_2_O^59^. Electrocompetent cells were kept on ice and used immediately for the recombineering reaction.

For recombineering, 50–150 ng of targeting DNA was electroporated at 18 kV cm^−1^ in 0.1 cm cuvettes using a micropulser (Biorad #165-210). Cells were immediately resuspended in 1 mL of SOC and then transferred to culture tubes containing 10 mL LB broth. After a 4 hour outgrowth at 30 °C, 100 µL of the culture was plated onto LB agar plates containing either 30 µg mL^−1^ carbenicillin or 30 µg mL^−1^ kanamycin. Colonies were checked for successful incorporation of recombinant DNA via colony PCR with oligos AD031 and AD032 for site A, AD022 and AD023 for site B, or AD024 and AD025 for site C (see Table S1).

### Purifying phage DNA from lysogens

Expression of recombinant phage was induced via heat shock. A single lysogenic colony was grown in 50 mL of LB broth with the appropriate antibiotic overnight at 30 °C. 5 mL of this starter culture was used to inoculate 500 mL of LB the following morning. When the flask reached an O.D._600_ ~ 0.6, the temperature was rapidly raised to 42 °C in a water bath. The culture was placed at 45 °C in a shaking incubator for 15 minutes and then transferred to a 37 °C incubator for two hours. To liberate the phage particles, cells were harvested by centrifugation at 3,000 RCF for 30 minutes and lysed via re-suspension in 10 mL of SM buffer (50 mM Tris-HCl [pH 7.5], 100 mM NaCl, 8 mM MgSO_4_) + 2% chloroform, and rotated at 37 °C for 30 min. A subsequent 1 hour incubation with 50 ng μL^−1^ DNaseI (Sigma# D2821) and 30 ng μL^−1^ RNaseA (Sigma# R6513) degraded the bacterial genomic DNA and RNA. The clarified lysate, containing soluble phage capsids, was obtained by centrifugation for 15 minutes at 6,000 RCF and 4 °C, and further diluted with 40 mL of SM buffer. Phage capsids were precipitated by incubating for 1 hour with 10 mL ice-cold buffer L2 (30% PEG 6000, 3 M NaCl) and then harvest by centrifugation at 10,000 RCF for 10 minutes at 4 °C. The phage pellet was washed with 1 mL of buffer L3 (100 mM Tris-HCl [pH 7.5], 100 mM NaCl, 25 mM EDTA) and then re-suspended with 3 mL of buffer L3, followed by an equal volume of buffer L4 (4% SDS). The phage capsid proteins were further digested by incubation with 100 ng μL^−1^ of proteinase K (NEB #P8012S) for 1 hour at 55 °C. SDS was precipitated with 3 mL buffer L5 (3 M potassium acetate [pH 7.5]), and the cloudy solution was clarified by centrifugation at 15,000 RCF for 30 minutes at 4 °C. The soluble phage DNA was passed over a pre-equilibrated Qiagen tip-500 column (Qiagen #10262), washed with buffer QC (1.0 M NaCl, 50 mM MOPS [pH 7.0], 15% isopropanol) and eluted with 15 mL buffer QF (1.25 M NaCl, 50 mM Tris [pH 8.5], 15% isopropanol). Finally, the DNA was precipitated with the addition of 10.5 mL of 100% isopropanol, rinsed in 70% ethanol and re-dissolved in TE buffer (10 mM Tris-HCl [pH 8.0], 1 mM EDTA) to a final DNA concentration of 200–500 ng μL^−1^. We routinely obtained ~250 µg of pure λ-DNA from a single purification.

### Inserting synthetic oligonucleotides into λ-DNA

Recombinant λ-DNA was obtained from strain IF189, which was modified and purified as described above, and 25 μg of the DNA was incubated with 150 U of Nt.BspQI (NEB# R0644S) in a 250 µL reaction with 1X buffer 3.1 (NEB #B7203) at 55 °C for 1 hour. The reaction was halted with 1 U of proteinase K (NEB #P8107S) for 1 hour at 55 °C. The nicked DNA was mixed with a 100-fold molar excess of the desired insert oligo (AD006 for 5′-flap, YK105 for (CAG)_13_, MB32 for mock insert, Table S1), along with a 10-fold excess of *cosL* and *cosR-*complementary oligos (IF003 and IF004 for *cosL* → *cosR*, IF001 and IF002 for *cosR* → *cosL*, Table S1). The solution was heated to 70 °C for 15 minutes followed by slow cooling to 22 °C in a thermocycler at a rate of −0.5 °C min^−1^. The annealed mixture was supplemented with 6000 U of T4 DNA ligase (NEB #M0202L) and 1 mM ATP and further incubated overnight at room temperature in a final reaction volume of 300 µL. A 50 μL aliquot was taken for alkaline agarose gel and restriction enzyme digest analysis. The remaining 250 µL was inactivated with high salt (1 M NaCl) and then passed through a 120 mL Sephacryl S-1000 column (GE #17-0476-01), in TE running buffer (10 mM Tris-HCl [pH 8.0], 1 mM EDTA) plus 150 mM NaCl, to separate the modified λ DNA from excess oligos and enzymes. Purified DNA was stored at 4 °C.

To verify incorporation of the insert, a restriction enzyme digests was performed with NcoI, an enzyme whose recognition site is abolished upon incorporation of the insert into our recombineered λ-DNA. 5 μL of the above mentioned ligation reaction was digested with 20 U of NcoI (NEB #R13193) in a 30 μL reaction with 1X Cutsmart buffer (NEB #B7204) for 1 hour at 37 °C and run on a 0.8% agarose gel stained with ethidium bromide. To check for complete ligation, a 5 µL aliquot was run over a 0.6% alkaline agarose gel at 15 mV for 20 hours at 4 °C. The gel was then submerged in neutralization solution (1 M Tris-HCl [pH 7.6], 1.5 M NaCl) for one hour, and stained in a solution of 10 mg mL^−1^ ethidium bromide for an additional hour. The stained gel was visualized using a Typhoon FLA 9500 laser scanner (GE).

### Imaging Lac repressor (LacI) on DNA curtains

To obtain distributions of triple FLAG epitope tagged LacI binding to recombineered Lac operator λ-DNA, we incubated 120 nM LacI with 10–25 ng μL^−1^ of biotinylated λ-DNA in a 150 μL volume of BSA buffer (40 mM Tris-HCl [pH 7.6], 50 mM NaCl, 2 mM MgCl_2_, 0.2 mg mL^−1^ BSA, 1 mM DTT) supplemented with 100 mM NaCl, for 5 min on ice. This mixture was diluted to a 1 mL final volume with BSA buffer and injected into flowcells, containing a streptavidin-activated lipid bilayer, to produce single-tethered DNA curtains bound with LacI at the Lac operator site. LacI was then fluorescently labeled *in situ* with an injection of 1.5 nM of anti-FLAG antibodies (Sigma-Aldrich #F9291) conjugated to fluorescent quantum dots (QDs, Life Tech #Q10163MP). The antibodies and QDs were pre-conjugated by mixing in a test tube for 10 minutes on ice prior to injection into the flowcell.

### Loading PCNA on DNA curtains

To observe the binding distribution of yPCNA on DNA, 0.8 nM yRFC was mixed with 1 nM yPCNA (concentrations reported for the trimer) in BSA buffer supplemented with 100 μM ATPγS (Roche). The yPCNA-yRFC(ATPγS) complex was injected into flowcells containing single-tethered DNA curtains, and incubated for 6 minutes at room temperature. Following this incubation, yPCNA was labeled *in situ* by injecting 1.5 nM of biotinylated anti-FLAG antibodies conjugated to fluorescent streptavidin-conjugated quantum dots (QDs, emitting at 705 nm, Thermo-Fisher). The antibodies and QDs were pre-conjugated in a test tube on ice prior to injection into the flowcell.

The same protocol was used to measure yPCNA diffusion dynamics with the flowing modifications. First, 1 mM ATP was used instead of 100 µM ATPγS during the yPCNA-yRFC(ATP) incubation. Second, 5 minutes after antibody-coupled QDs were injected into the flowcell, residual yRFC and free QDs were washed out by injecting 200 μL of BSA buffer +300 mM NaCl over 1 minute. yPCNA diffusion data was acquired for 10 minutes after this yRFC washing step.

### Single-Molecule Microscopy

DNA curtains were assembled in custom-made flowcells consisting of a microscope slide patterned with 1–2 µM-wide Chromium barriers to lipid diffusion. These features were deposited using standard UV lithography, as described previously^32^. A jeweler’s drill press (Servo Products, Model #7110) and diamond-plated bits (Jewelry Tools, Product ID DIB-211.00) were used to drill two inlet ports along the long face of the quartz slides. Next, 5 mm-wide channels were cut in double-sided sticky tape (3 M #665) and sandwiched between the microfabricated quartz slide and a glass coverslip (#50-948-951, Fisher Scientific). This sandwich was held together with binder clips and baked at 150 °C for 1 hour. Finally, nanoports (IDEX #N-333) were glued to the drilled side of the assembled flowcell using a hobbyist hot glue gun (Superbonder, #HE-750).

Single-molecule fluorescence images were collected with a Nikon Ti-E microscope in a prism-TIRF configuration. The inverted microscope setup allowed for the sample to be illuminated by a 488 nm laser light (Coherent) through a quartz prism (Tower Optical Co.). The laser was adjusted to deliver 40 mW of power at the front face of the prism. Experiments were conducted on a floating TMC optical table to avoid spatial drift. A 60x water immersion objective lens (1.2 NA, Nikon), two EM-CCD cameras (Andor iXon DU897, −80 °C) and NIS-Elements software (Nikon) were used to collect the data with a 50 ms exposure time. Two-color imaging was conducted using a 638 nm dichroic beam splitter (Chroma). Frames were saved as TIFF files without compression for further image analysis in FIJI, a distribution of the open-source ImageJ software (NIH)^60^.

### Data Analysis

#### Particle tracking

Fluorescent particles were tracked in FIJI with a custom-written particle tracking script (available upon request)^60^. The resulting trajectories were analyzed in MATLAB R2014a (Mathworks). The fluorescent particle in each frame was fit to a two-dimensional Gaussian function to obtain its position with sub-pixel resolution. The series of positions of a given particle were used to obtain trajectories^56, 61^.

yPCNA-binding distributions were determined by calculating the distance between the center of DNA-bound fluorescent particles and the chromium barrier. yPCNAs were deemed to be on the DNA substrate if they retracted to the chromium barrier when buffer flow was toggled off and on. All binding distributions were collected from at least three flowcells. When indicated, these were fit to a Gaussian distribution using the MATLAB curve-fitting toolbox.

#### Statistical methods

Error bars on the yPCNA binding distributions were calculated in MATLAB using bootstrap analysis with replacement^62^. Statistical tests were conducted in the PAST3 software package^63^ or in MATLAB (Mathworks). A Pearson’s chi-squared test was used to determine whether the different DNA substrates have statistically significant effects on yPCNA sliding (Tables S2 and S3). The significance threshold was set at 0.05 in all tests.

Wild type yRFC nonspecifically loads yPCNA on homoduplex DNA^43^. This makes it difficult to determine whether the mild enrichment at the (CAG)_13_ repeat is statistically significant for any single target DNA. Therefore, we used the two-tailed t test to determine whether loading yPCNA at the (CAG)_13_ repeat on all three DNA substrates has the same normalized frequency as loading on all homoduplex DNA sequences (the null hypothesis). For this test, the entire DNA substrate was divided into eight windows, each containing 5 kb of DNA. A 5 kb window captures >99% of all target-bound yPCNAs within our spatial resolution, as determined by analysis of the 5′-ssDNA flap structure (Fig. 4). Thus, each modified DNA substrate has a single window with a (CAG)_13_ repeat and 7 windows with nonspecific homoduplex DNA. A two-tailed t-test demonstrated that the mild enrichment in the (CAG)_13_ window is statistically significant relative to yPCNA binding in all other homoduplex windows (Fig. S3).

#### Measuring diffusion coefficients

*y*PCNA diffusion coefficients were measured on double-tethered DNA curtains that extend DNA molecules between two micro-fabricated chromium features in the absence of buffer flow^32, 34^. The average separation between the two chromium features was 13 µm (~80% extension relative to B-form DNA). Trajectories of individual molecules were used to calculate the one-dimensional (1D) mean squared displacement (MSD) as a function of time interval using:

1 $$MSD(n{\rm{\Delta }}t)=\frac{1}{N-n}\,\sum _{i=1}^{N-n}{({y}_{i+n}-{y}_{i})}^{2}$$

where *N* is the total number of frames in the trajectory, n is the number of frames for a given time interval, Δ*t* is the time between frames, and *y*_*i*_ is the yPCNA position at frame *i*. The MSD was calculated for the first 10 time intervals (Δ*t* = 0.05 s to 0.5 s) and plotted as a function of Δ*t* to generate the line:

2 $$MSD({\rm{\Delta }}t)=2D{\rm{\Delta }}t$$

where *D* is the diffusion coefficient. Plots were fit to a line in MATLAB and the slope was used to calculate diffusion coefficients of individual yPCNA molecules. Diffusion coefficients were calculated for ≥30 molecules per NaCl concentration, and are reported as a mean ± standard error (S.E.).

## Electronic supplementary material

Supplementary Figures, Tables, and References
