# Supplementary material for: Efficient modification of λ-DNA substrates for single-molecule studies
Source: Sci Rep. 2017 May 18;7:2071. doi: 10.1038/s41598-017-01984-x (PMC5437064; doi:10.1038/s41598-017-01984-x)
Supplement: Supplementary file 1 — Supplementary Figures, Tables, and References [file 41598_2017_1984_MOESM1_ESM.pdf]

Supplementary information for:

Efficient modification of λ-DNA substrates for single-molecule studies

Yoori Kim[1], Armando de la Torre[1], Andrew A. Leal[1], and Ilya J. Finkelstein[1,2,*]

[1]Department of Molecular Biosciences and Institute for Cellular and Molecular Biology, The University of Texas at Austin, Austin, Texas 78712

[2]Center for Systems and Synthetic Biology, The University of Texas at Austin, Austin, Texas 78712
* To whom correspondence should be addressed. Tel: 1-512-471-1394; e-mail: ifinkelstein@cm.utexas.edu

Keywords: DNA curtains, recombineering, single-molecule imaging, phage, nicking enzyme

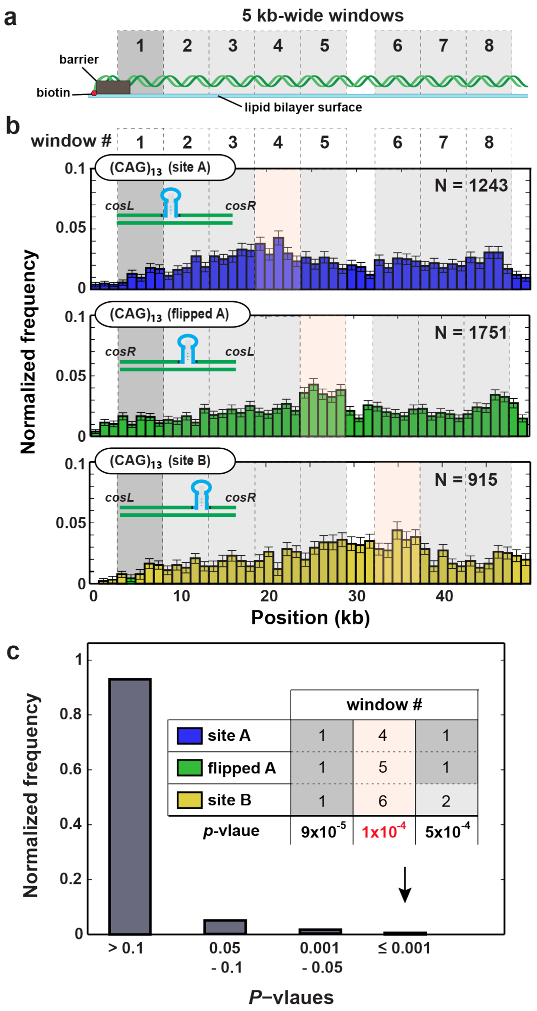

Figure S1. yRFC has a mild preference for loading yPCNA at $(CAG)_{13}$ repeats relative to homoduplex dsDNA. (a) To determine whether the mild yPCNA-yRFC(ATPγS) enrichment at the $(CAG)_{13}$ was statistically significant, the DNA substrate was divided into eight 5 kb-wide windows (dashed lines). The first window (dark gray box) is partially obstructed by the Cr barriers. Windows containing the $(CAG)_{13}$ repeat are shown in pink. (b) Normalized binding distribution of yPCNA-yRFC(ATPγS) complexes on three DNA substrates containing a $(CAG)_{13}$. The binding histograms were also divided into 5 kb-wide windows (dashed lines). The window partially obscured by the Cr barrier is marked in dark gray, homoduplex DNA is gray, and the target-containing window is pink. A two-tailed t-test was used to compare the groupings of three windows—including the three $(CAG)_{13}$-containing window—against all homoduplex DNA windows. (c) A normalized histogram of all two-tailed t-tests comparing the mean yPCNA occupancy of all three-window combinations relative to the mean yPCNA occupancy in all windows containing homoduplex DNA. Over 97% of the tests showed no significance (p-value > 0.05). The highest p-values are shown in the inset and include the $(CAG)_{13}$-containing sites, as well as the partially obstructed first window. This is because the Cr barrier causes the first window to underestimates yPCNA binding. All p-values in the p=0.001-0.05 range were from window groupings that included two of three $(CAG)_{13}$ repeat windows or window #1.

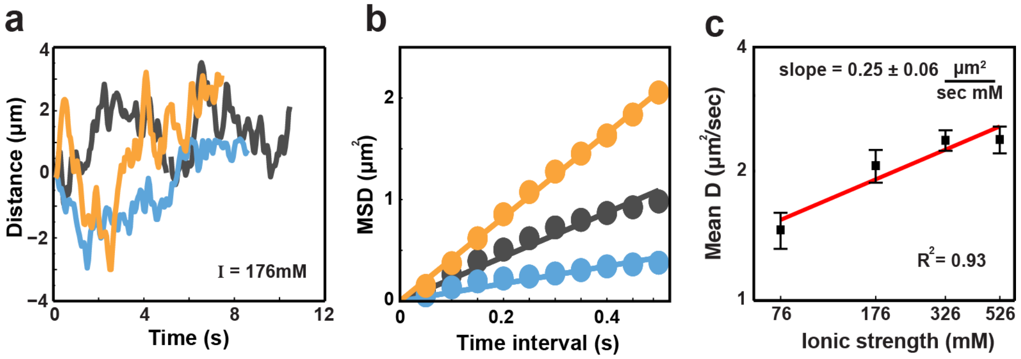

Figure S2. Characterization of yPCNA diffusion on homoduplex λ-DNA. (a) Representative single-molecule traces of the position of individual yPCNA molecules (shown in three different colors) on double-tethered DNA curtains at a total ionic strength I = 176 mM. (b) Mean squared displacement (MSD) of each of the molecules in (a). The MSDs are fit to a line, and the slopes are used to calculate the one-dimensional diffusion coefficients (solid lines). (c) Mean yPCNA diffusion coefficients as a function of the ionic strengths (error bars: S.E.M; to N = 30, 29, 29, 31 for 76 mM, 176 mM, 326 mM, and 525 mM ionic strengths, respectively). The red line indicates a linear fit through the data with a slope of 0.25 ± 0.06 μm^2 (sec mM)$^{-1}$. The error in the slope represents the standard error of the fit. These results are consistent with a single-molecule study that looked at hPCNA diffusion on homoduplex DNA[1]. Diffusion of both hPCNA and yPCNA was weakly dependent on the ionic strength (0.33 ± 0.04 μm^2 sec^{-1} mM^{-1} and 0.25 ± 0.06 μm^2 sec^{-1} mM^{-1} for hPCNA and yPCNA, respectively).

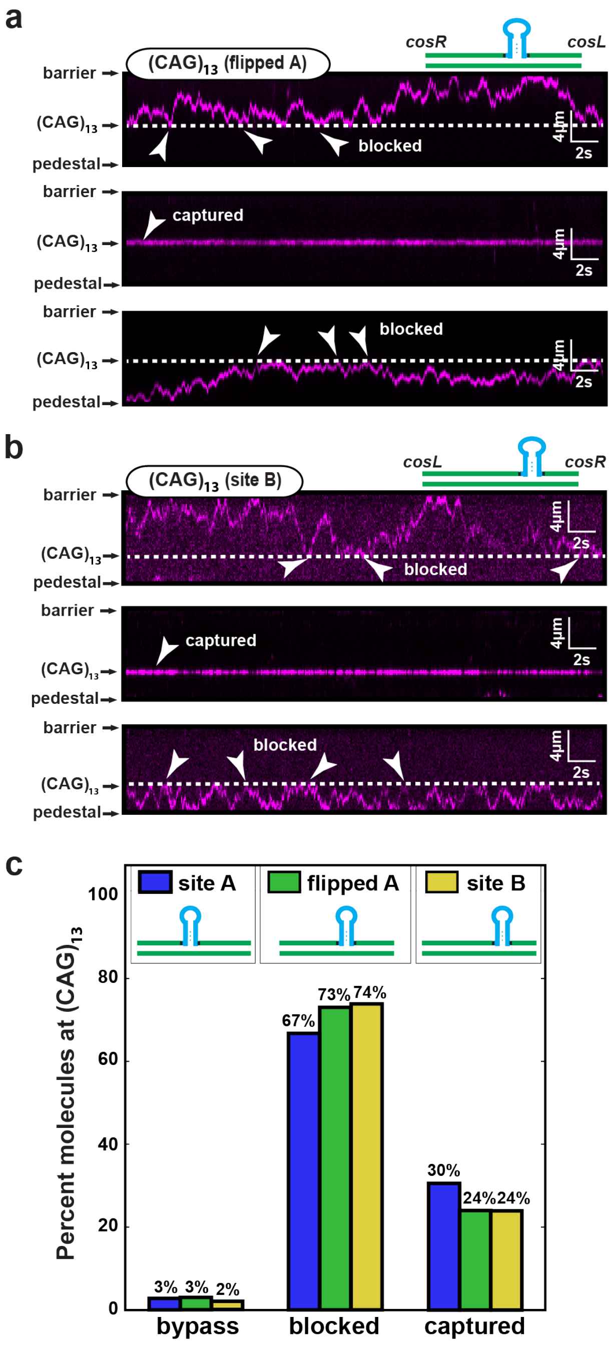

Figure S3. yPCNA diffusion on DNA substrates containing $(CAG)_{13}$ repeats. (a) Representative kymographs of diffusing yPCNA on DNA having $(CAG)_{13}$ at flipped site A or at (b) site B. Dashed lines indicate the position of a $(CAG)_{13}$ repeat. Arrows: yPCNA is blocked by the $(CAG)_{13}$ structure. (c) Percentage of molecules showing either bypass, blocked, or captured behavior at $(CAG)_{13}$ inserts. At least 35 DNA molecules were analyzed and classified into each of three categories (N=36, 62, and 46 for site A (from Fig. 5d), flipped site A, and site B). A chi-squared test shows that the distribution of yPCNA behaviors at each of the three $(CAG)_{13}$-containing DNAs is statistically indistinguishable (p-value = 0.95).

Supplemental DNA Sequences

Complete DNA sequences for each of the six plasmids in Figure 1 are also available via Benchling: https://benchling.com/ifinkelstein/

Legend:
bold: homology for λ-DNA
orange: LacO
blue: Kanamycin resistance cassette
green: three BspQI sites
restriction sites: NcoI is double-underlined and NotI site is single-underlined.

Recombineering cassette for site A

CGGGAATGATCCAGATTTTGCTACCACCATGACTAACGCGCTTGCGGGTAAACAACCGAAGAATGCGACAC
TGACGGCGCTGGCAGGGCTTTCCACGGCGAAAAATAAATTACCGTATTTTGCGGAAAATGATGCCGCCAGC
CTGACTGAACTGACTCAGGTTGGCAGGGATATTCTGGCAAAAAATTCCGTTGCAGATGTTCTTGAATACCT
TGGGGCCGGTGAGAATTCGGCCTTTCCGGCAGGTGCGGAGCATTGCTACGGCGATTCCTAGAATTGTGAGC
GCTCACAATTCTAGTTGGAGAGCCACTGTTCATTTAAATAAAGCTCTTCATGCATGCGGCCGCTCTTCCCA
TGGTGCGATCGCTCTTCGGGATTTAAATAGGTACCTATGGACAGCAAGCGAACCGGAATTGCCAGCTGGGG
CGCCCTCTGGTAAGGTTGGGAAGCCCTGCAAAGTAAACTGGATGGCTTTCTTGCCGCCAAGGATCTGATGG
CGCAGGGGATCAAGATCTGATCAAGAGACAGGATGAGGATCGTTTCGCATGATTGAACAAGATGGATTGCA
CGCAGGTTCTCCGGCCGCTTGGGTGGAGAGGCTATTCGGCTATGACTGGGCACAACAGACAATCGGCTGCT
CTGATGCCGCCGTGTTCCGGCTGTCAGCGCAGGGGCGCCCGGTTCTTTTTGTCAAGACCGACCTGTCCGGT
GCCCTGAATGAACTGCAGGACGAGGCAGCGCGGCTATCGTGGCTGGCCACGACGGGCGTTCCTTGCGCAGC
TGTGCTCGACGTTGTCACTGAAGCGGGAAGGGACTGGCTGCTATTGGGCGAAGTGCCGGGGCAGGATCTCC
TGTCATCTCACCTTGCTCCTGCCGAGAAAGTATCCATCATGGCTGATGCAATGCGGCGGCTGCATACGCTT
GATCCGGCTACCTGCCCATTCGACCACCAAGCGAAACATCGCATCGAGCGAGCACGTACTCGGATGGAAGC
CGGTCTTGTCGATCAGGATGATCTGGACGAAGAGCATCAGGGGCTCGCGCCAGCCGAACTGTTCGCCAGGC
TCAAGGCGCGCATGCCCGACGGCGAGGATCTCGTCGTGACCCATGGCGATGCCTGCTTGCCGAATATCATG
GTGGAAAATGGCCGCTTTTCTGGATTCATCGACTGTGGCCGGCTGGGTGTGGCGGACCGCTATCAGGACAT
AGCGTTGGCTACCCGTGATATTGCTGAAGAGCTTGGCGGCGAATGGGCTGACCGCTTCCTCGTGCTTTACG
GTATCGCCGCTCCCGATTCGCAGCGCATCGCCTTCTATCGCCTTCTTGACGAGTTCTTCTGA**TTTCGTCAT**
GTTTTGAGTCTGCTGTTGATATTTCTAAAGTCGGTTTTTTTTCTTCGTTTTCTCTAACTATTTTCCATGAA
ATACATTTTTGATTATTATTTGAATCAATTCCAATTACCTGAAGTCTTTCATCTATAATTGGCATTGTATG
TATTGGTTTATTGGAGTAGATGCTTGCTTTTCTGAGCCATAGCTCTGATATCCAAATGAAGCCATAGGCAT
TTGTTATTTTGGCTCTGTCAGCTGCATA

Recombineering cassette for site B

TTTAGTCTGGATAGCCATAAGTGTTTGATCCATTCTTTGGGACTCCTGGCTGATTAAGTATGTCGATAAGG
CGTTTCCATCCGTCACGTAATTTACGGGTGATTCGTTCAAGTAAAGATTCGGAAGGGCAGCCAGCAACAGG
CCACCCTGCAATGGCATATTGCATGGTGTGCTCCTTATTTATACATAACGAAAAACGCCTCGAGTAGTGCA
GCCCTAGAATTGTGAGCGCTCACAATTCTAGACTCGATGACGCGGCCGCTATGGACAGCAAGCGAACCGGA
ATTGCCAGCTGGGGCGCCCTCTGGTAAGGTTGGGAAGCCCTGCAAAGTAAACTGGATGGCTTTCTTGCCGC
CAAGGATCTGATGGCGCAGGGGATCAAGATCTGATCAAGAGACAGGATGAGGATCGTTTCGCATGATTGAA
CAAGATGGATTGCACGCAGGTTCTCCGGCCGCTTGGGTGGAGAGGCTATTCGGCTATGACTGGGCACAACA
GACAATCGGCTGCTCTGATGCCGCCGTGTTCCGGCTGTCAGCGCAGGGGCGCCCGGTTCTTTTTGTCAAGA
CCGACCTGTCCGGTGCCCTGAATGAACTGCAGGACGAGGCAGCGCGGCTATCGTGGCTGGCCACGACGGGC
GTTCCTTGCGCAGCTGTGCTCGACGTTGTCACTGAAGCGGGAAGGGACTGGCTGCTATTGGGCGAAGTGCC
GGGGCAGGATCTCCTGTCATCTCACCTTGCTCCTGCCGAGAAAGTATCCATCATGGCTGATGCAATGCGGC
GGCTGCATACGCTTGATCCGGCTACCTGCCCATTCGACCACCAAGCGAAACATCGCATCGAGCGAGCACGT
ACTCGGATGGAAGCCGGTCTTGTCGATCAGGATGATCTGGACGAAGAGCATCAGGGGCTCGCGCCAGCCGA
ACTGTTCGCCAGGCTCAAGGCGCGCATGCCCGACGGCGAGGATCTCGTCGTGACCCATGGCGATGCCTGCT

TGCCGAATATCATGGTGGAAAATGGCCGCTTTTCTGGATTCATCGACTGTGGCCGGCTGGGTGTGGCGGAC
CGCTATCAGGACATAGCGTTGGCTACCCGTGATATTGCTGAAGAGCTTGGCGGCGAATGGGCTGACCGCTT
CCTCGTGCTTTACGGTATCGCCGCTCCCGATTCGCAGCGCATCGCCTTCTATCGCCTTCTTGACGAGTTCT
TCTGAGCTAGC**AATTAATGTGCATCGATTATCAGCTATTGCCAGCGCCAGATATAAGCGATTTAAGCTAAG**
AAAACGCATTAAGATGCAAAACGATAAAGTGCGATCAGTAATTCAAAACCTTACAGAAGAGCAATCTATGG
TTTTGTGCGCAGCCCTTAATGAAGGCAGGAAGTATGTGGTTACATCAAAACAATTCCCATACATTAGTG

Recombineering cassette for site C

ACATCAAAGCAGTCTGTCAGTCAGTGCGTGAAGCCACCACCGCCTCCGGCGTGGATAATGCAGCCTCCCCC
CGACTGGCAGACACCGCTGAACGGGATTATTTCACCCTCAGAGAGAGGCTGATCACTATGCAAAAACAACT
GGAAGGAACCCAGAAGTATATTAATGAGCAGTGCAGATAGAGTTGCCCATATCGATGGCTCGAGTAGTGCA
GCCCTAGAATTGTGAGCGCTCACAATTCTAGACTCGATGACGCGGCCGCTATGGACAGCAAGCGAACCGGA
ATTGCCAGCTGGGGCGCCCTCTGGTAAGGTTGGGAAGCCCTGCAAAGTAAACTGGATGGCTTTCTTGCCGC
CAAGGATCTGATGGCGCAGGGGATCAAGATCTGATCAAGAGACAGGATGAGGATCGTTTCGCATGATTGAA
CAAGATGGATTGCACGCAGGTTCTCCGGCCGCTTGGGTGGAGAGGCTATTCGGCTATGACTGGGCACAACA
GACAATCGGCTGCTCTGATGCCGCCGTGTTCCGGCTGTCAGCGCAGGGGCGCCCGGTTCTTTTTGTCAAGA
CCGACCTGTCCGGTGCCCTGAATGAACTGCAGGACGAGGCAGCGCGGCTATCGTGGCTGGCCACGACGGGC
GTTCCTTGCGCAGCTGTGCTCGACGTTGTCACTGAAGCGGGAAGGGACTGGCTGCTATTGGGCGAAGTGCC
GGGGCAGGATCTCCTGTCATCTCACCTTGCTCCTGCCGAGAAAGTATCCATCATGGCTGATGCAATGCGGC
GGCTGCATACGCTTGATCCGGCTACCTGCCCATTCGACCACCAAGCGAAACATCGCATCGAGCGAGCACGT
ACTCGGATGGAAGCCGGTCTTGTCGATCAGGATGATCTGGACGAAGAGCATCAGGGGCTCGCGCCAGCCGA
ACTGTTCGCCAGGCTCAAGGCGCGCATGCCCGACGGCGAGGATCTCGTCGTGACCCATGGCGATGCCTGCT
TGCCGAATATCATGGTGGAAAATGGCCGCTTTTCTGGATTCATCGACTGTGGCCGGCTGGGTGTGGCGGAC
CGCTATCAGGACATAGCGTTGGCTACCCGTGATATTGCTGAAGAGCTTGGCGGCGAATGGGCTGACCGCTT
CCTCGTGCTTTACGGTATCGCCGCTCCCGATTCGCAGCGCATCGCCTTCTATCGCCTTCTTGACGAGTTCT
TCTGAGCTAGC**CTCTGGAAGCATTCAGAGCAATTGAGGCAGCGTTGGTGAAGCACGATAATAATATGAAGG**
ATTATTCCCTGGTGGTTGACTGATCACCATAACTGCTAATCATTCAAACTATTTAGTCTGTGACAGAGCCA
ACACGCAGTCTGTCACTGTCAGGAAAGTGGTAAAACTGCAACTCAATTACTGCAATGCCCTCGTAATTAA

Nickase cassette

ATTTAAATAAAGCTCTTCATGCATGCGGCCGCTCTTCCCATGGTGCGATCGCTCTTCGGGATTTAAATA

Table S1. Oligonucleotides used in this study

Name	Sequence
AD006	**TTTTTTTTTTTTTTTTTTTTTTTTTTTTTT**TCTTCCCATGGTGCGATCGC TCTTCG
YK105	/5Phos/TGCATGCGGCCGCTCTTCCT**CAGCAGCAGCAGCAGCAGCAGCA GCAGCAGCAGCAGCAG**ATGGTGCGATCGCTCTTCG
MB032	/5Phos/TGCATGCGGCCGCTCTTCCCATGGTGCGATCGCTCTTCG
AD012	AGTCTGGATAGCCATAAGTG
AD013	GTAACCACATACTTCCTGCC
AD016	GCAGTCTGTCAGTCAGTGCG
AD017	CGAGGGCATTGCAGTAATTG
AD022	CGTTCATGGCTGAACTCCTG
AD023	CGGCATAACATGCAGTGGAC
AD024	GTGATGTTGCTGCGCTCGATG
AD025	CTCAGCCTGGGTCATTGAAG
AD027	GCTACCACCATGACTAACGC
AD028	GGATATCAGAGCTATGGCTC
AD031	CCGCGATTGCAGATGTTATC
AD032	CTATACAGCCAAGCTTGCAG
IF001	/5Phos/GGGCGGCGACCT/3BioTEG
IF002	/5Phos/AGGTCGCCGCCC/3Dig_N
IF003	/5Phos/AGGTCGCCGCCC/3BioTEG
IF004	/5Phos/GGGCGGCGACCT/3Dig_N
YK_PC NA01	/5phos/GATGACGACAAGCATCATCATCAT
YK_PC NA02	GTCTTTGTAGTCGCTGCTGCTGCCCATGGTAT

** The nucleotides for 5'-ssDNA flap or $(CAG)_{13}$ are shown in **bold**.
** The complementary nucleotides to λ-DNA are shown in blue.

References

1. Kochaniak, A. B. *et al*. Proliferating cell nuclear antigen uses two distinct modes to move along DNA. *J Biol Chem* **284,** 17700–10 (2009).
